# Tumor-targeted delivery of a C-terminally truncated FADD (N-FADD) significantly suppresses the B16F10 melanoma via enhancing apoptosis

**DOI:** 10.1038/srep34178

**Published:** 2016-10-21

**Authors:** Yun-Wen Yang, Chun-Mei Zhang, Xian-Jie Huang, Xiao-Xin Zhang, Lin-Kai Zhang, Jia-Huang Li, Zi-Chun Hua

**Affiliations:** 1 The State Key Laboratory of Pharmaceutical Biotechnology, School of Life Sciences, Nanjing University, Nanjing, 210023, Jiangsu, China; 2 Changzhou High-Tech Research Institute of Nanjing University and Jiangsu TargetPharma Laboratories Inc., Changzhou, 213164, Jiangsu, China

## Abstract

Fas-associated protein with death domain (FADD), a pivotal adaptor protein transmitting apoptotic signals, is indispensable for the induction of extrinsic apoptosis. However, overexpression of FADD can form large, filamentous aggregates, termed death effector filaments (DEFs) by self-association and initiate apoptosis independent of receptor cross-linking. A mutant of FADD, which is truncated of the C-terminal tail (m-FADD, 182–205 aa) named N-FADD (m-FADD, 1–181 aa), can dramatically up-regulate the strength of FADD self-association and increase apoptosis. In this study, it was found that over-expression of FADD or N-FADD caused apoptosis of B16F10 cells *in vitro*, even more, N-FADD showed a more potent apoptotic effect than FADD. Meanwhile, Attenuated *Salmonella Typhimurium* strain VNP20009 was engineered to express FADD or N-FADD under the control of a hypoxia-induced NirB promoter and each named VNP-pN-FADD and VNP-pN-N-FADD. The results showed both VNP-pN-FADD and VNP-pN-N-FADD delayed tumor growth in B16F10 mice model, while VNP-pN-N-FADD suppressed melanoma growth more significantly than VNP-pN-FADD. Additionally, VNP-pN-FADD and VNP-pN-N-FADD induced apoptosis of tumor cells by activating caspase-dependent apoptotic pathway. Our results show that N-FADD is a more potent apoptotic inducer and VNP20009-mediated targeted expression of N-FADD provides a possible cancer gene therapeutic approach for the treatment of melanoma.

Apoptosis is an active, genetically controlled process and is important for homeostasis of the body^1^,^2^,^3^. There are two major pathways involves in apoptosis, the intrinsic and extrinsic apoptotic pathway^2^,^4^. Fas associated protein with death domain (FADD), composed of two structurally similar motifs, an N-terminal death effector domain (DED) and a C-terminal death domain (DD), is a key adaptor protein mediating death receptor signals from the plasma membrane to the cytoplasm in extrinsic apoptotic pathway^5^. FADD transmits apoptotic signals from all known DRs such as Fas^6^, tumor necrosis factor receptor 1(TNF-R1), ligand-receptor (TRAIL-R1) (DR4), TRAIL-R2 (DR5) and so on^7^,^8^. FADD associates with the DRs through its DD, whereas the cytoplasmic procaspase-8 or procaspase-10 binds to FADD through DED-DED interactions^9^,^10^. In addition to its adaptor function mediating the apoptotic signal form extracellular death receptor, over-expression of FADD can form large, filamentous aggregates, termed death effector filaments (DEFs) by self-association and then initiate apoptosis independent of receptor for FADD aggregates serving as a platform can recruit and activate procaspase-8 efficiently and autoinitiate apoptosis^11^,^12^,^13^. Our previous studies have shown that a mutant of human FADD, which is truncated of the C-terminal tail (h-FADD, 183–208 aa) named N-FADD (h-FADD, 1–182 aa), can dramatically up-regulate the strength of FADD self-association observed by fluorescence resonance energy transfer (FRET)^14^.

Melanoma is an aggressive form of cancer, and the reduced levels of apoptosis in some melanoma were found due to upregulation of antiapoptotic molecules such as Flice-inhibitory protein (c-FLIP)^15^ or downregulation of DcR1 and DcR2^16^. As apoptosis deficiency resulting in therapy resistance of melanoma, it is highly suggestive for restoring and strengthening apoptosis in treatment of melanoma^2^,^17^, for example, Venza *et al*. found the histone deacetylase inhibitor MS-275 synergized with TRAIL could induce apoptosis in TRAIL-resistant cell lines by decreasing c-FLIP which providing a promising therapeutic approach for treatment of melanoma^18^. On the other hand, low expression or degradation of the FADD adaptor protein affords apoptosis deficiency in some tumor cells^19^,^20^. Over-expression of FADD or its variants can self-induced apoptosis of tumor cells, making the potential of FADD or its variants used as anti-tumor agents. However, systemic administration of FADD with low tumor targeting delivery may have some side effects. Therefore, targeted delivery of FADD or its variants into tumor cells determines their antitumor efficacy.

*Salmonella typhimurium* is one of facultative anaerobic gram-negative bacteria can selectively accumulate and replicate in solid tumors for the existence of hypoxic areas and immune-privileged environment within these tumors^21^,^22^. Attenuated *S. typhimurium* VNP20009 is one of the most popular strains used for tumor therapy for its low toxicity, high tumor targeting, genetically stability, and antibiotic susceptibility^23^,^24^. Several studies showed that VNP20009 mediated anticancer agents such as endostatin^25^, TRAIL^26^, CCL21^27^, HPRG^28^ and SPRY^29^ can suppress tumor growth and prolong survival time in murine tumor models. Engineered plasmids or proteins carried by recombinant *S. typhimurium* can be released into tumor cells or phagocytic cells^30^,^31^. The characteristic of *S. typhimurium* makes it possible for targeted delivery of protein into tumor cells. Although the mechanism of bacterial cancer therapy has yet to be understood, *S. typhimurium* induced the pyroptosis and apoptosis of tumor cells enhancing its therapeutic efficacy^32^,^33^. For the apoptosis deficiency in tumor cells, we hypothesized that tumor-specific delivery of FADD or its variant by VNP20009 with the potential to enhance apoptosis and improve the antitumor effect of VNP20009.

As described above, a C-terminally truncated FADD (N-FADD) shows an enhanced ability of evoking apoptosis. However, the previous results were based on FRET at the single-cell level and the anti-tumor efficacy of N-FADD was not known^14^. In the present study, a mutant of mouse FADD (m-FADD, 1–181 aa), which is truncated of the C-terminal tail (m-FADD, 182–205 aa) similar to human N-FADD (h-FADD, 1–182 aa), was constructed and VNP20009 was engineered to express FADD or N-FADD (VNP-pN-FADD, VNP-pN-N-FADD) under the control of a hypoxia-induced NirB promoter in tumor tissues. Our group has analyzed and reported that the proteins under control of NirB promoter was specifically expressed in tumor tissue but couldn’t be detected in the normal tissues, avoiding potential toxicity to normal tissues^28^,^29^,^34^. After intraperitoneal injection of genetically engineered *Salmonella* strains into mice carrying melanoma xenografts, it was found that VNP-pN-N-FADD significantly suppressed tumor growth and prolonged survival time of mouse, suggesting a potential strategy for melanoma therapy.

## Results

### Over-expression of FADD or N-FADD induced apoptosis of B16F10 melanoma through activating caspase-3

**Figure 1:**
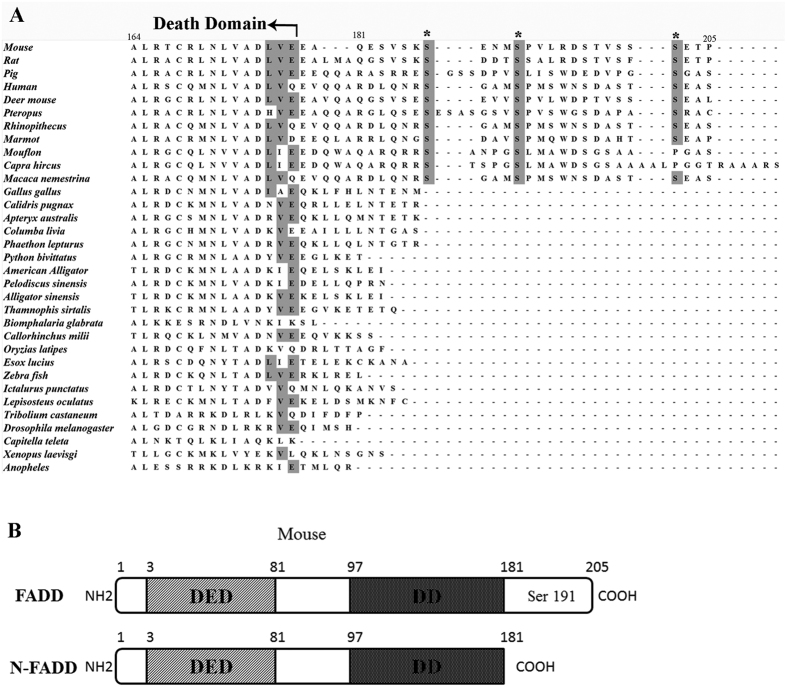
Alignment of FADD proteins from different species and diagram of N-FADD. (**A**) Alignment of FADD proteins from several different species. Only the C-terminal regions are shown to highlight the conservation of the known phosphorylation sites, ser 191 of mouse FADD (equivalent to Ser194 of human FADD), and the other two putative ones, Ser 187, Ser 202, are indicated by asterisks to highlight their conservation in mammals. The residue numbers are based on the corresponding amino acid residue position in the mouse FADD protein. All of the FADD protein sequences were obtained from NCBI and aligned by Clustalw. (**B**) FADD and N-FADD of mouse. The numbers indicate the amino acid residue position in the mouse FADD protein.

As a well-known adaptor protein, FADD can transmit apoptotic signals from members of the tumor necrosis factor receptor family. However, without receptor cross-linking, over-expression of FADD can initiate cell apoptosis by self-association, forming large, filamentous aggregates, termed death effector filaments (DEFs)^11^. The death domain (DD) and death effector domain (DED) of FADD are essential for interaction with death receptors and transmission of the apoptotic signal, while the DED and DD are highly conserved across all species examined (Fig. 1A). Interestingly, there is a conserved C-terminal region in mammals (Fig. 1A) while the C-terminal region is absent in lower organisms such as Zebra fish, Xenopus laevisg, Anopheles and Drosophila indicating the C-terminal region was acquired during evolution^35^. The C-terminal region of the mammalian FADD plays non-apoptotic functions such as in cell cycle regulation, regulation of protein kinase C inactivation and so on^35^,^36^,^37^. Even more, our previous studies showed that a mutant of human FADD, which is truncated of the C-terminal tail (h-FADD, 183–208 aa) named N-FADD (h-FADD, 1–182 aa), can dramatically up-regulate the strength of FADD self-association and increase cell apoptosis^14^. In this study, we constructed a mutant of mouse FADD which is truncated of the C-terminal tail (m-FADD, 182–205 aa) named N-FADD (m-FADD, 1–181 aa) (Fig. 1B) similar to human N-FADD (h-FADD, 1–182 aa).

**Figure 2:**
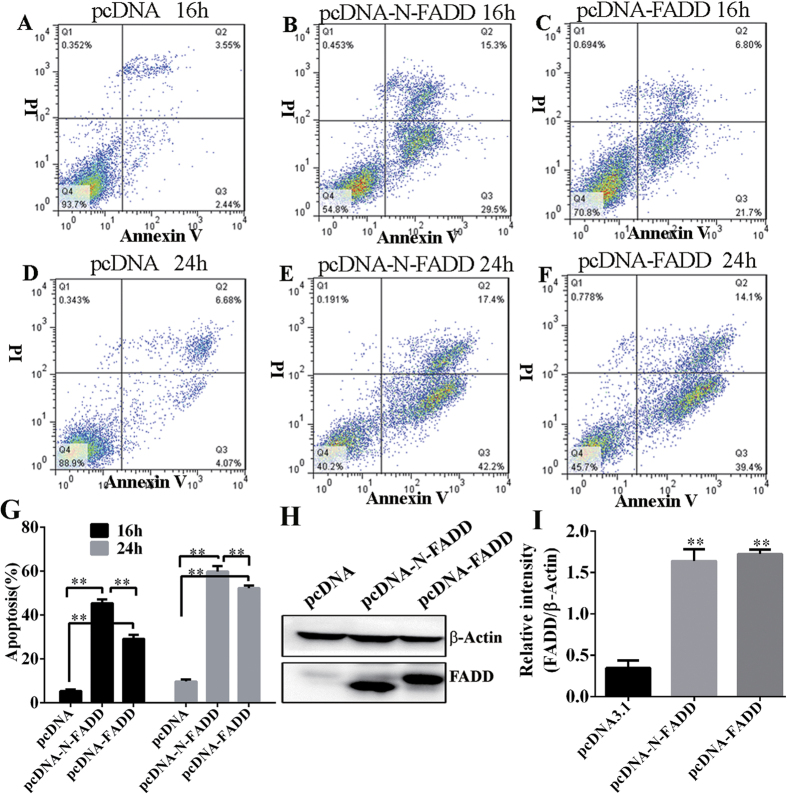
Overexpression of FADD or N-FADD induced apoptosis of B16F10 melanoma cells *in vitro*. (**A–C**) Representative FACS analysis of Annexin V and propidium iodide (PI) staining after transfection of FADD or N-FADD expressing or empty vectors for 16 h. (**D**–**F**) Representative FACS analysis of Annexin V and PI staining after transfection of FADD or N-FADD expressing or empty vectors for 24 h. (**G**) Overexpression of FADD or N-FADD induced apoptosis of B16F10 melanoma cells (Mean ± SD, n = 3 independent experiments); **P < 0.01. (**H**) Western blots analysis of expression of FADD after transfection of FADD or N-FADD expressing or empty plasmids in B16F10 cells. β-actin was served as loading control. (**I**) Statistical analysis of western blot by Image J; **P < 0.01, compared with pcDNA3.1. One representative of three independent experiments is displayed.

Firstly, we checked whether over-expression of mouse FADD and its truncated variant N-FADD (m-FADD, 1–181 aa) in B16F10 melanoma cells could induce the apoptosis of the cells. After transfecting equal amount of pcDNA3.1 (−), pcDNA-FADD and pcDNA-N-FADD respectively, the apoptosis of B16F10 cells were analyzed by flow cytometry at different time points. As shown in Fig. 2, over expression of FADD or N-FADD evoked the B16F10 melanoma cells apoptosis significantly compared with empty vector pcDNA3.1 (−) (Fig. 2G). FADD and its variant N-FADD increased the cell death as extension of overexpression time, N-FADD caused about 45% cell death compared to 28% of FADD after 16 hours’ transfection (Fig. 2B,C), while the gap between FADD and N-FADD was reduced as the time prolonged when post transfection for 24 h, FADD induced about 53% cells death to 59% of N-FADD (Fig. 1E,F). However, N-FADD showed a more potent induction of cell apoptosis than FADD (Fig. 2G). Immunoblotting was constructed to analyze overexpression of FADD and N-FADD in B16F10 cells (Fig. 2H). Statistical analysis of western blot shows the amount of FADD or N-FADD was the same (Fig. 2I).

**Figure 3:**
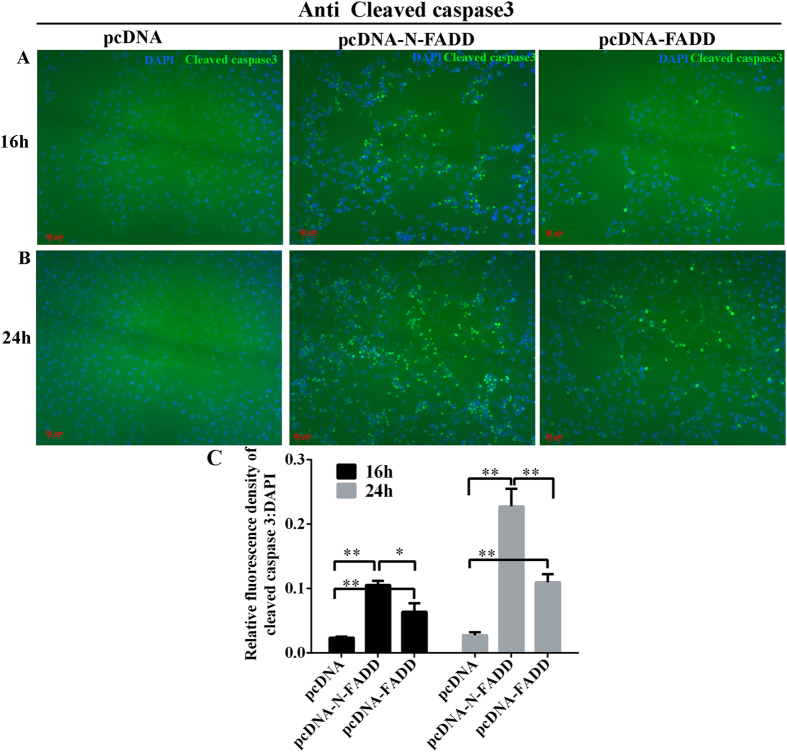
Overexpression of FADD or N-FADD in B16F10 melanoma cells activation of caspase-3. (**A**) Representative immunofluorescence staining for cleaved caspase-3 (green) after transfection of FADD or N-FADD expressing or empty vectors for 16 h. (**B**) Representative immunofluorescence staining for cleaved caspase-3 (green) after transfection of FADD or N-FADD expressing or empty vectors for 24 h. (**C**) Quantification analysis of relative cleaved caspase3 expression level (fluorescence intensity of cleaved caspase3/DAPI) by Image J software after transfecting FADD or N-FADD expressing or empty vectors, *P < 0.05, **P < 0.01. Data are expressed as mean ± SD of three independent experiments.

Siegel *et al*. previously reported that FADD protein has the potential to form protein aggregates serving as a platform for procaspase-8 recruitment and activation^11^. Activated caspase-8 then directly cleaves pro-caspase-3 or other executioner caspases, eventually leading to the apoptosis. In our experiment, immunofluorescence and its quantitative results showed transfection of FADD or N-FADD caused apoptosis of B16F10 melanoma cells by activating pro-caspase-3 (Fig. 3). As shown in Fig. 3, overexpression of FADD or N-FADD in B16F10 cells can activate caspase-3 and even more, N-FADD can significantly increase cleaving of pro-caspase-3 compared with wild type FADD both after 16 h or 24 h transfection. In all, overexpression of FADD or N-FADD induced apoptosis of B16F10 cells through activating caspases-dependent cell apoptosis pathway.

### Construction and characterization of the recombinant *Salmonella* strains for tumor-specific delivery of FADD and N-FADD

**Figure 4:**
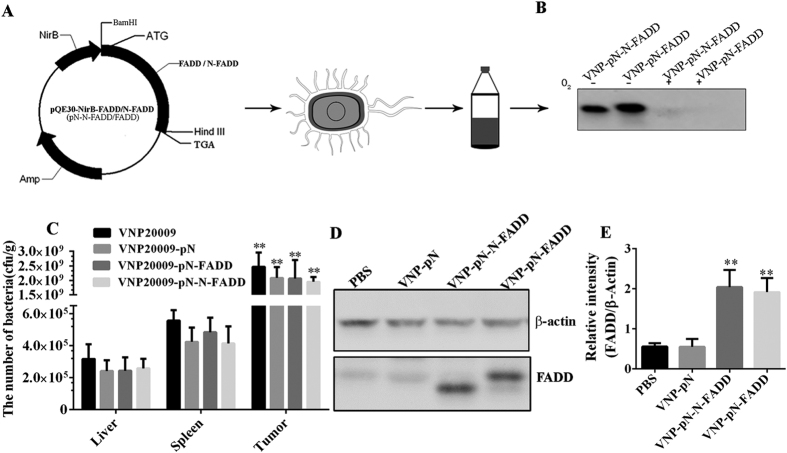
FADD and N-FADD delivered by *S. typhimurium* strain VNP20009 expressed *in vitro* and *in vivo* under the control of NirB promoter. (**A**) pQE30-NirB-FADD and pQE30-NirB-N-FADD plasmids were constructed and transformed into VNP (VNP-pN-FADD and VNP-pN-N-FADD). VNP-pN-FADD and VNP-pN-N-FADD were grown for 72 h under anaerobic or aerobic conditions. Bacterial lysates were subjected to immunoblotting assays using anti-FADD (N term) antibodies (**B**). (**C**) The relative tissue distribution of indicated stable strains was detected by colony formation assay. *p < 0.001, tumor compared with the other tissues. Data are expressed as mean ± SD of five animals. (**D**) Detection of the expression of FADD or N-FADD in tumor tissues of the mice bearing melanoma by western blotting. β-actin was served as loading control. (**E**) Statistical analysis the results of western blot by Image J; **P < 0.01, compared with PBS group. One representative of three independent experiments is displayed.

The above results demonstrated that an effective induction apoptosis of B16F10 melanoma cells was achieved by transfecting FADD or N-FADD. Next, we try to tumor-specifically deliver FADD and N-FADD *in vivo*. VNP20009 has been proved as a promising tumor-target vector for tumor therapy as VNP20009 can specifically accumulate and replicate in the tumors. In order to engineer the VNP20009 for tumor-specific delivery of FADD and N-FADD, the coding sequences of mouse FADD and its variant N-FADD were cloned into the plasmid pQE30-NirB (Fig. 4A), in which the expression of the recombinant proteins was driven under the control of a hypoxia-inducible NirB promoter. Then the appropriate plasmids were transformed into the VNP20009, and the stable recombinant strains were selected by ampicillin (100 μg/ml).

In order to analyze the expression of FADD and N-FADD *in vitro*, the stable strains were cultured under aerobic and anaerobic conditions, respectively. Western blot showed that FADD and N-FADD proteins were expressed exclusively in anaerobic conditions (Fig. 4B). To determine the bacterial biodistribution and analyze the capability of NirB promoter to express exogenous proteins in hypoxic tumor regions, the recombinant strains VNP, VNP-pN, VNP-pN-FADD and VNP-pN-N-FADD were injected into mice bearing B16F10 melanoma xenograft respectively. Three days after intraperitoneal injection of the appropriate strains, mice bearing melanoma were sacrificed and the tissues were removed and homogenized for analysis of bacteria titer. Our previous studies showed that engineered VNP20009 strains mainly accumulated in tumor, spleen and liver, while the bacteria could hardly be detected in the other tissues, such as heart, lung and kidney^28^,^29^. In this study, colony formation assays revealed that all the strains preferentially accumulated in tumors compared with spleens or liver tissues (P < 0.001) (Fig. 4C). After treatment for 4 days, the number of appropriate strains in tumor tissues reached 1.5–3.0 × 10^9^ cfu/g compared to 3.0–6.5 × 10^5^ cfu/g in spleens and 1.0–4.5 × 10^5^ cfu/g in liver tissues. Although carrying plasmids declined tumor target of VNP slightly, there were no significant differences among VNP, VNP-pN, VNP-pN-FADD and VNP-pN-N-FADD *in vivo* distribution, suggesting that the expression of exogenous proteins didn’t significantly affect the growth and tumor targeting abilities of VNP20009 (P > 0.05). Next, we examined whether the recombinant proteins were successfully expressed *in vivo*. Western blotting results showed that FADD and N-FADD proteins were expressed in tumor tissues treated by VNP-pN-FADD or VNP-pN-N-FADD, but were not detected in the VNP and VNP-pN groups, indicating that the NirB-driven expressions of exogenous FADD and N-FADD were successfully expressed at the same level in tumor tissues (Fig. 4D,E). It must be noticed that the expression of exogenous proteins under control of NirB promoter was so weaken in normal tissues that couldn’t be detected by western blotting (data not show). Briefly, using this strategy, we successfully make tumor-specific delivery of FADD and N-FADD proteins.

### *S. typhimurium* strain-mediated tumor-targeted delivery of N-FADD significantly suppresses tumor growth and enhances survival time in B16F10 mice model

**Figure 5:**
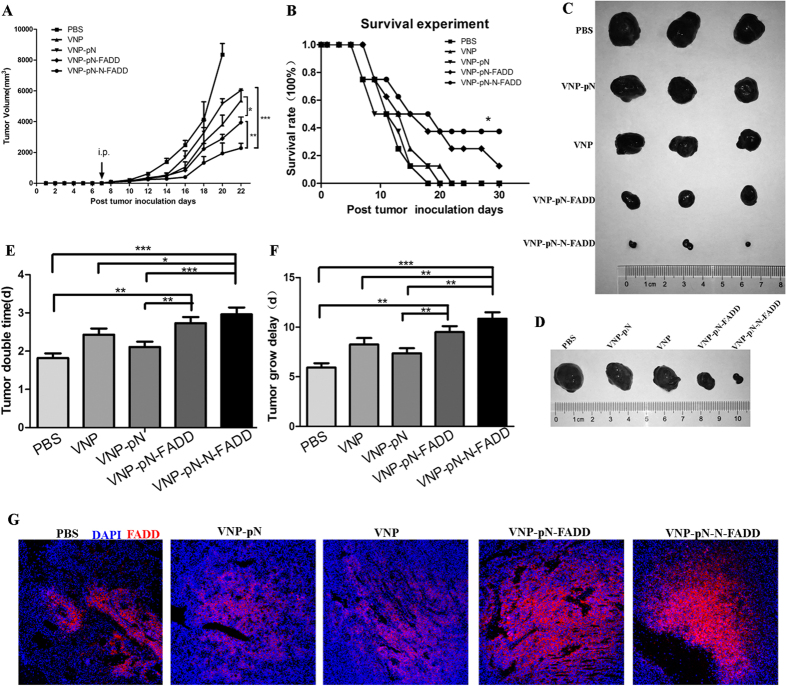
VNP-pN-N-FADD delayed tumor growth and enhanced survival time in B16F10 mice model. (**A**) Tumor growth curves of groups as indicated. B16F10 tumor mice per group (n = 10 mice) were injected i.p. with 2 × 10^5^ cfu of VNP, VNP-pN, VNP-pN-FADD and VNP-pN-N-FADD or with PBS at day 7. Tumor volumes among different groups were compared. Data are presented as mean ± SD. *P < 0.05 for VNP-pN-FADD versus VNP or VNP-pN; **P < 0.01 for VNP-pN-N-FADD versus VNP-pN-FADD; ***P < 0.001 for VNP-pN-N-FADD versus VNP or VNP-pN. (**B**) Kaplan-Meier survival curves of mice bearing B16F10 melanomas. Data were analyzed by the log-rank test. *P < 0.05 for VNP-pN-N-FADD versus PBS, VNP, VNP-pN and VNP-pN-FADD. (**C,D**) Representative therapeutic efficacy of the recombinant VNP strains for melanoma therapy. (**E,F**) Tumor doubling time and growth delay. Data are presented as mean ± SD, *P < 0.05, **P < 0.01, ***P < 0.001, (n = 10 mice). (**G**) Immunofluorescence staining of FADD (stained red), the representative images (100×) revealing FADD or N-FADD expression in B16F10 tumor tissue treated with PBS, VNP, VNP-pN, VNP-pN-FADD and VNP-pN-N-FADD.

Since the engineered VNP strains could preferentially accumulate and express the genes of interest in the hypoxic tumor regions, we further examined the tumor suppressing efficacy of recombinant strains VNP-pN-FADD and VNP-pN-N-FADD in B16F10 mice model. Seven days after the establishment of the melanoma mouse model and when the volumes of tumor were 100 mm^3^ to 150 mm^3^, mice were treated with appropriate VNP strains or PBS control by intraperitoneal injection. Tumor volumes were recorded for the evaluation of the therapeutic efficacy. As shown in Fig. 5A, the tumor volumes of VNP, VNP-pN, and VNP-pN-FADD, VNP-pN-N-FADD groups were significantly lower than that of PBS group. Compared with VNP and VNP-pN groups, the tumor volume in both VNP-pN-FADD mice (P < 0.05) and VNP-pN-N-FADD mice (P < 0.001) significantly decreased (Fig. 5A). Furthermore, VNP-pN-N-FADD showed significantly inhibited melanoma growth compared with VNP-pN-FADD (P < 0.01). The Kaplan-Meier survival assay showed that the cumulative survival rate and median survival time in VNP-pN-N-FADD mice were significantly increased compared with that in PBS, VNP, VNP-pN and VNP-pN-FADD mice (log-rank tests, P < 0.05) (Fig. 5B). Unexpectedly, VNP-pN-FADD did not significantly prolonged survival time of tumor-bearing mice compared with VNP and VNP-pN (P > 0.05). After treatment with engineered VNP strains, the tumor doubling time was prolonged from 1.82 d (CI, 1.71 d to 1.93 d) in the PBS control group, 2.40 d (CI, 2.25 d to 2.59 d) (CI: Confidence Interval) in VNP group, or 2.10 d (CI, 1.98 d to 2.24 d) in VNP-pN group to 2.70 d (CI, 2.55 d to 2.88 d) in VNP-pN-FADD and 2.95 d (CI, 2.79 d to 3.15 d) in VNP-pN-N-FADD (Fig. 5E). However, VNP-pN-FADD did not significantly prolonged the tumor doubling time compared with VNP (P > 0.05). Tumor growth delay also increased from 5.94 d (CI, 5.50 d to 6.37 d) in PBS control group, 8.27 d (CI, 7.60 d to 8.90 d) in VNP group, or 7.37 d (CI, 6.86 d to 7.88 d) in VNP-pN group to 9.50 d (CI, 8.90 d to 10.10 d) in VNP-pN-FADD group and 10.85 d (CI, 10.20 d to 11.50 d) in VNP-pN-N-FADD (P < 0.05) (Fig. 5F).

Moreover, immunofluorescence stained with anti-FADD (N-term) antibody revealed that the recombinant proteins were preferentially expressed in the necrotic areas of the tumors (Fig. 5G), suggesting that FADD and N-FADD proteins were efficiently expressed in tumor hypoxia regions. Therefore, the above results reinforce our attempt that *Salmonella*-mediated expression of N-FADD in tumor tissues is a possible efficient and safety strategy for melanoma therapy.

### VNP-pN-N-FADD yields its therapeutic effects in melanoma xenografts by inducing apoptosis of tumor cells through activating caspase-3 dependent cell death

**Figure 6:**
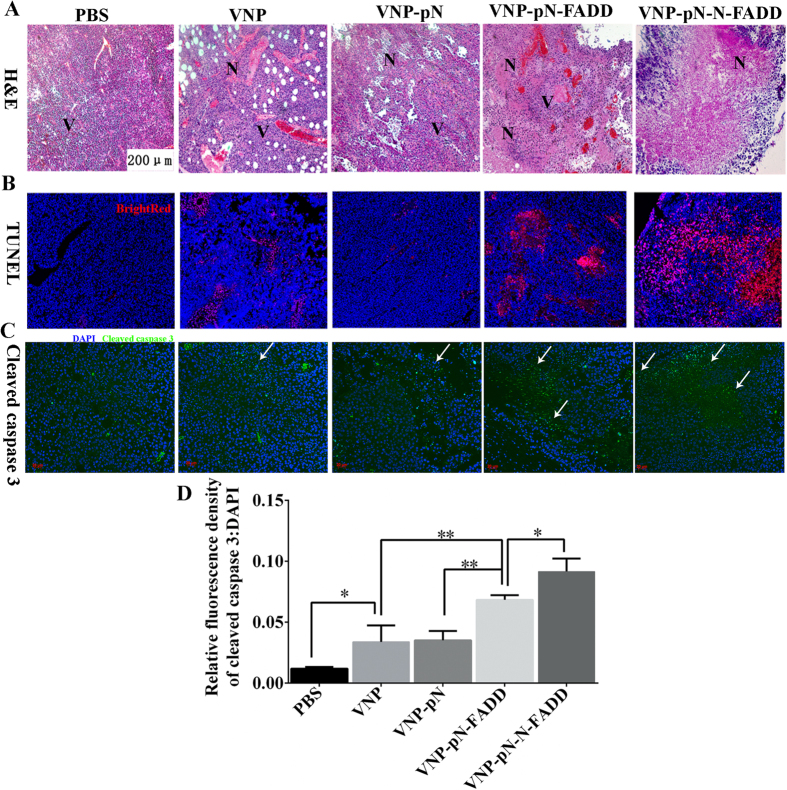
*Salmonella typhimurium* strain VNP20009 carrying FADD or N-FADD induced apoptosis of melanoma cells. (**A**) H&E staining of the tumor sections. The representative images (100×) revealing necrotic areas of B16F10 tumor tissue treated with PBS, VNP, VNP-pN, VNP-pN-FADD and VNP-pN-N-FADD; N, necrotic tumor regions; V, vital tumor regions. (**B**) TUNEL assay of melanoma. The representative images (100×) revealing apoptosis of tumor cells treated with appropriate VNP strains; bright red, apoptotic cells. (**C**) Immunofluorescence staining of cleaved caspase-3 (stained green and indicated with blank arrows), the representative images (200×) revealing cleaved caspase-3 in B16F10 tumor tissue treated with PBS, VNP, VNP-pN, VNP-pN-FADD and VNP-pN-N-FADD. (**D**) Quantification analysis of relative cleaved caspase-3 expression level (fluorescence intensity of cleaved caspase-3 DAPI) by Image J software, *P < 0.05, **P < 0.01, bar represents the mean ± SD of five optical fields. Each experiment was carried out in triplicate.

As described above, overexpression of FADD or its variant N-FADD self-initiates apoptosis of B16F10 melanoma cells by activating caspases dependent cell death *in vitro*. In order to examine if overexpression of FADD or N-FADD in melanoma tumor tissues could enhance the apoptosis of tumor cells, the mice treated with appropriate recombinant VNP strains for 12 days were sacrificed and frozen tissue sections (5 μm in thickness) were prepared according to standard protocols. H & E staining (Fig. 6A) showed all of VNP, VNP-pN, VNP-pN-FADD and VNP-pN-N-FADD could lead to formation of tumor necrosis areas, while VNP-pN-FADD and VNP-pN-N-FADD caused much more necrosis areas than VNP and VNP-pN. Even more, VNP-pN-N-FADD induced the most serious cell death and formed the biggest necrosis areas among all of recombinant VNP strains. Additionally, TUNEL assays were carried out and the results showed that a greater number of tumor cells underwent apoptosis in mice treating with VNP-pN-FADD and VNP-pN-N-FADD compared with mice receiving VNP or VNP-pN (Fig. 6B), suggesting that FADD or N-FADD overexpression in tumor tissues could induce more melanoma cells to undergo apoptosis and VNP-pN-N-FADD induced the most number of tumor cells to undergo apoptosis among all of the recombinant VNP strains. Furthermore, immunofluorescence staining of cleaved caspase-3 in tumor sections indicated that treatment with VNP-pN-FADD or VNP-pN-N-FADD greatly activated caspase-3 compared with VNP or VNP-pN (p < 0.01), even though both VNP and VNP-pN also significantly activated caspase-3 compared with PBS control (p < 0.05) (Fig. 6C,D). Meanwhile, VNP-pN-N-FADD showed a potent ability of inducing cleavage of caspase-3 more than VNP-pN-FADD (p < 0.05) (Fig. 6D). Briefly, our experiments indicated that VNP-pN-FADD and VNP-pN-N-FADD likely suppressed tumor growth and improved survival of melanoma-bearing mice by inducing apoptosis of tumor cells through caspase-dependent cell death pathway, and VNP-pN-N-FADD induced more cleavage of caspase-3 than VNP-pN-FADD, thus VNP-pN-N-FADD displaying a more potent antitumor efficacy than VNP-pN-FADD.

## Discussion

Resistance to apoptosis or de-regulated apoptotic signaling, particularly the activation of an anti-apoptotic systems in tumor, allows tumor cells to escape apoptosis leading to uncontrolled proliferation resulting in tumor survival, then resulting in therapeutic resistance and recurrence of tumor^2^,^17^,^38^. Fas associated protein with death domain (FADD) and its variant N-FADD aggregate and autoinitiate the apoptosis signal and induce tumor cells death^11^,^13^ makes them as possible anti-tumor agents.

Several facultative anaerobe strains, including *Clostridium*^39^, *Bifidobacterium*^40^, *Listeria*^41^ and *S. typhimurium*^42^,^43^, exhibited significant potential in cancer therapy for their characteristic of preferential accumulation in the hypoxic region of solid tumors^44^. Attenuated *S. typhimurium* VNP20009 is an outstanding antitumor agent for the inhibition of tumor growth; meanwhile, recent studies have showed the efficacy of VNP in delivering many kinds of therapeutic agents to the tumor site^28^,^29^,^31^. However, successful bacterial antitumor therapy, in which facultative anaerobe strains are used to express anticancer proteins, should overcome the obstacles as follows. Firstly, the antitumor protein should be expressed selectively at the tumor site for a small number of bacteria may replicate in normal tissues, posing potential toxicity to normal tissues^45^,^46^. Our previous studies showed that a hypoxia-induced promoter NirB drives the expression of genes of interest only under anaerobic conditions both *in vitro* and *in vivo*^28^,^29^,^34^. In this study, the NirB promoter was designed to express FADD or N-FADD protein in tumor tissue. As shown in Fig. 4B,D, FADD and N-FADD proteins were successfully expressed under control of NirB promoter *in vitro* and *in vivo*. Secondly, the release of antitumor proteins also affects anti-tumor efficacy of engineered strains. It should be noticed that most of antitumor proteins act on the cancer cell surface or the tumor microenvironment, or on intracellular targets. For effective release of antitumor proteins act on intracellular targets, *Salmonella* type III secretion system or cell-penetrating peptides (CPPs) was widely used as a strategy^29^,^47^. However, not all of antitumor proteins delivered by *S. typhimurium* were applicable with type III secretion system or cell-penetrating peptides (CPPs) for secretion peptides or CPPs, which might alter the normal structure and functions of therapeutic proteins to some extent. Although *S. typhimurium* is found to survive in membrane bound vacuoles when internalized, after invading into tumor cells or phagocytic cells, some of bacteria are destroyed, then the anti-tumor proteins expressed by recombinant *S. typhimurium* can be released into cytosol^30^,^31^. FADD mainly exist and play its apoptotic roles in the cytosol^5^. Auto initiate apoptosis needs FADD forming large, filamentous aggregates, termed death effector filaments (DEFs) by self-association and then serving as a platform to efficiently recruit and activate procaspases^11^,^14^. In order to keep the normal structure and self-association functions of FADD protein or its variant, we did not utilize any secretion peptides or CPPs in this study. Although antitumor proteins expressed and delivered by attenuated *S. typhimurium* without any secretion peptides or CPPs may influence the efficiency release of the proteins into tumor cells more or less, our results showed that N-FADD delivered by VNP under the control of NirB promoter (VNP-pN-N-FADD) was successfully expressed in tumor sites and significantly suppressed the growth of tumor and prolonged survive time of tumor-bearing mice compared with other engineered strains (P < 0.05) (Fig. 5). However, it must be noted FADD delivered by VNP (VNP-pN-FADD) significantly suppressed the growth of tumor compared with VNP and VNP-pN groups (P < 0.05), but did not significantly prolong survive time of the melanoma-bearing mice compared with VNP and VNP-pN groups (P > 0.05) (Fig. 4B).

*Salmonella* could induce apoptosis and suppressed the growth of solid tumor when used as a single antitumor agent^32^. In this study, we demonstrated that nirB promoter-driven expression of FADD (VNP-pN-FADD) or its variant N-FADD (VNP-pN-N-FADD) by VNP could markedly suppressed melanoma growth, even more, VNP-pN-N-FADD with a more potent anti-tumor efficacy and markedly extended the survival of mice bearing melanoma than VNP-pN-FADD (Fig. 5). TUNEL assays showed that the antitumor effects of FADD or N-FADD were mainly mediated through evoking and enhancing apoptosis of tumor cells (Fig. 6B). Immunofluorescence staining of cleaved caspase-3 showed that VNP-pN-N-FADD induction more caspase-3 cleavage than VNP-pN-FADD (P < 0.05) (Fig. 6C), suggesting that overexpression of N-FADD induced more cell death than wild type FADD through enhancing caspase dependent cell apoptosis pathway.

In conclusion, our study first shows that N-FADD is a potential anti-tumor agent by enhancing apoptosis of tumor cells, providing an effective and promising strategy for the melanoma gene therapy, even though further studies are needed to evaluate its potentiality in the clinical application.

## Material and Methods

### Bacterial Strains, Cells and Animals

The eukaryotic expression vector pcDNA3.1 (−) was purchased from Invitrogen (Invitrogen, USA). Plasmids pcDNA-FADD and pQE30-NirB were maintained in our laboratory. *Salmonella Typhimurium* VNP20009 (*msbB*−/*purI*−) was obtained from ATCC (USA) and cultured in modified Luria-Bertani (LB) media at 37 °C. *Salmonella typhimurium* VNP20009 (*msbB*−/*purI*−) was abbreviated to VNP. B16F10 melanoma cells were purchased from American Type Culture Collection (ATCC, USA). Cells were cultured at 37 °C in 5% CO_2_ in a humidified atmosphere in Dulbecco’s modified Eagle’s media (DMEM, Gibco, Shanghai, China) supplemented with 10% fetal bovine serum (FBS, Gibco, Australia), penicillin (100 IU/ml) and streptomycin (100 μg/ml). Five-to six-week-old female C57BL/6 mice were obtained from Model Animal Research Center of Nanjing University (Nanjing, China), and maintained in pathogen-free conditions for one week before the start of the experiment. This study was approved by the Animal Care and Use Committee of Nanjing University and was carried out in accordance with the Guide for the Care and Use of Laboratory Animals by the National Research Council.

### Plasmids Construction and Bacterial Transformation

N-FADD (m-FADD, 1–181 aa), which is truncated of the C-terminal tail of mouse FADD, was amplified by primers N-FADD-F and N-FADD-R with pcDNA-FADD as template. Primers sequences as follows:

N-FADD-F: 5′-CG*GGATCC*ATGGACCCATTCCTGGTGC-3′

N-FADD-R: 5′-CCC*AAGCTT*TCACTGGGCTTCTTCCACCAG-3′

The sequence was cloned into eukaryotic expression vector pcDNA3.1 (−) after digested with *BamHI* and *HindIII*. The positive clone contains sequence of N-FADD named pcDNA-N-FADD. For hypoxia-inducible expression in VNP, the sequences of FADD and N-FADD were inserted into the prokaryote expression vector pQE30-NirB with *BamHI*, *HindIII* digestion and the resultant vectors were named pQE-NirB-FADD and pQE-NirB-N-FADD, respectively. All of the coding sequences of positive clones were confirmed to be correct by DNA sequencing (GenScript Corporation, Nanjing, Jiangsu, China). pQE-NirB, pQE-NirB-FADD and pQE-NirB-N-FADD plasmids were transformed into VNP using a Gene Pulser apparatus (Bio-Rad, Hercules, CA, USA) with conditions as follows: 2.5 kV, 25 μF and 400 Ω, respectively and plated on LB agar containing 100 μg/ml ampicillin. Positive VNPs were each named VNP-pN, VNP-pN-FADD and VNP-pN-N-FADD.

### Expression of FADD and N-FADD *in vitro*, and colony formation assays

In order to analyze the function of NirB promoter, 2 × 10^9^ colony-forming units (CFU) of VNP-pN-FADD or VNP-pN-N-FADD were grown at 37 °C to the logarithmic phase and was inoculated into 50 ml of LB media and cultured under aerobic and anaerobic conditions. The anaerobic LB medium was prepared by boiling to remove dissolved oxygen. Residual oxygen was driven out further by nitrogen for 15 min in boiling water. Then 0.05% sodium sulfide and 0.05% cysteine were added to maintain the reducing environment in anaerobic jars (Oxid, London, UK). After 3 days, VNP-pN-FADD and VNP-pN-N-FADD were harvested by centrifugation at 4 °C and suspended in 1 ml of phosphate buffer saline (PBS) buffer. After repeated sonication, the solution was centrifuged at 12000 rpm, 10 min, 4 °C, and the supernatant was collected. The expression of FADD or N-FADD was analyzed by western blot. For colony formation assays, the number of CFU was determined as previously described^48^.

### Annexin V /PI apoptosis assay

B16F10 melanoma cells were plated into 24-well plate. When the cell density reached to 60–70%, equal amount (0.5 μg/well) of pcDNA-FADD or pcDNA-N-FADD plasmids were transfected into cell by PolyJet™ reagent (Signagen, USA) according to the manufacturer’s instructions. After over expression for 16 h or 24 h, cells were harvested by trypsinization and washed with binding buffer, then stained with EGFP-conjugated-Annexin V for 15 min on ice in the dark. Each sample was added 1 μL PI and mixed gently before flow cytometry analysis (BD PharMingen, SanDiego, CA, USA). Triplicate experiments were performed in a parallel manner for each time point. Data were analyzed using FlowJo analysis software (Tree Star, Ashland, OR, USA).

### Construction of Mouse Tumor Model and antitumor studies

B16F10 melanoma cells, grown in DMEM with 10% FBS, were collected and suspended in PBS (pH 7.4). Cell concentration was adjusted to 2 × 10^6^ cells/ml, then 100 μl of cells (2 × 10^5^ cells) was injected subcutaneously into the hind flank region of the C57BL/6 mice. The tumors were allowed to grow. When the volume of tumor was about 100 mm^3^ to 150 mm^3^, the mice were randomly divided, then intraperitoneal injection of 2 × 10^5^ cfu VNP, VNP-pN, VNP-pN-FADD and VNP-pN-N-FADD per mouse, respectively and injection of PBS as control. The length and width of the tumor were measured every two days using a Vernier caliper (Mytutoyo Corporation, Japan) across its two perpendicular diameters. The number and dates of death of mice were recorded to calculate the survival rate. Tumor volume was determined using the formula: tumor volume = length × width^2^ × 0.52. Animal studies designed to maintain a high standard of animal welfare were approved by the Animal Care and Use Committee of Nanjing University.

### Western blot analysis

Cells were seeded at 4 × 10^5^ per well in a six-well plate for 12 hours, then plasmids pcDNA-FADD, pcDNA-N-FADD and pcDNA3.1 (−) were transfected by PolyJet™ reagent (Signagen, USA) following the manufacturer’s instructions. Following transfection for 24 h, to obtain whole-cell extracts, cells were washed with cold 1 × PBS, scrapped and disrupted with 100 μL of lysis buffer: 50 mM Tris-HCl (pH 7.4), 250 mM NaCl, 0.5% Triton X-100, 50 mM NaF, 2 mM EDTA and 1 mM Na3VO4supplemented with 1× protease inhibitor cocktail (Roche, Germany). After 30 minutes of incubation on ice, whole-cell extracts were cleared by centrifugation. Protein concentration was calculated with the BCA method (Pierce, USA), then 40 to 60 μg total protein were used for western blot analysis following standard conditions with primary antibody: Diluted 1:1000 for FADD (N-term) (Epitomics, 1825-1, USA), 1:1000 for actin (Abgent, Suzhou, China) and detected with ECL plus Western blot detection system (Tanon, Shanghai, China). Additional, tumor tissues were homogenized in lysis buffer and incubated on ice for 30 min; the supernatant was used for western blot after centrifugation. Bacterial lysates were prepared from VNP20009 carrying appropriate vectors as described above. Intensity was quantitatively analyzed by Image J software (NIH, Bethesda, MD, USA).

### Hematoxylin and Eosin (H&E) Staining, TUNEL assays, and Immunofluorescence Microscopy

After treatment for 12 days, mice in PBS, VNP, VNP-pN, VNP-pN-FADD and VNP-pN-N-FADD groups were sacrificed. Frozen tumor sections (5 μm in thickness) were prepared according to standard protocols for H&E staining. TUNEL assays were carried out by detecting apoptotic nuclei using the TUNEL BrightRed Apoptosis Detection Kit as instructed by the manufacturer (Vazyme, Nanjing, China). In order to analyze the activation level of caspase-3, the tumor sections were stained with anti-cleaved caspase-3 (Cell Signaling Technology, USA). In addition, immunofluorescence was conducted to analyze the expression of FADD or N-FADD in tumor tissues by staining with anti FADD (N-term) antibody (Epitomics, 1825-1, USA). The secondary antibodies for cleaved caspase-3 and FADD were goat anti-Rabbit IgG labeled with FITC (Invitrogen, USA). The relative fluorescence intensity was analyzed by Image J software.

### Statistical Analysis

The results are presented as Mean ± SD. Data were analyzed using Graph Pad Prism version 6.0 (Graph Pad Software, San Diego, CA, USA). The effect of treatment on survival time was determined using the log-rank test. Paired Student’s t-test analysis was conducted to assess statistical significance. P < 0.05 was considered to indicate a statistically significant difference.

## Additional Information

**How to cite this article**: Yang, Y.-W. *et al*. Tumor-targeted delivery of a C-terminally truncated FADD (N-FADD) significantly suppresses the B16F10 melanoma via enhancing apoptosis. *Sci. Rep.* **6**, 34178; doi: 10.1038/srep34178 (2016).
